# Racial disparities in emergency mental healthcare utilization among birthing people with preterm infants

**DOI:** 10.1016/j.ajogmf.2021.100546

**Published:** 2021-12-04

**Authors:** Kayla L. Karvonen, Rebecca J. Baer, Bridgette Blebu, Lucia Calthorpe, Jonathan D. Fuchs, Laura Jelliffe-Pawlowski, Deborah Karasek, Safyer McKenzie-Sampson, Scott P. Oltman, Larry Rand, Maureen T. Shannon, Taylor E. Washington, Tiana Woolridge, Elizabeth E. Rogers, Matthew S. Pantell

**Affiliations:** Department of Pediatrics, University of California San Francisco, San Francisco, CA; California Preterm Birth Initiative, San Francisco, CA; University of California San Diego, La Jolla, CA; Department of Obstetrics, Gynecology, and Reproductive Sciences, University of California San Francisco, San Francisco, CA; University of California San Francisco School of Medicine, San Francisco, CA; San Francisco Department of Public Health, San Francisco, CA; Departments of Epidemiology and Statistics; Family Health Care Nursing, University of California San Francisco, San Francisco, CA; Tulane University, New Orleans, LA.

**Keywords:** emergency department, neonatal intensive care unit, parental leave, postpartum depression, prematurity, preterm hospitalization, rehospitalizations, structural racism

## Abstract

**BACKGROUND:**

Birthing people of color are more likely to deliver low birthweight and preterm infants, populations at significant risk of morbidity and mortality. Birthing people of color are also at higher risk for mental health conditions and emergency mental healthcare utilization postpartum. Although this group has been identified as high risk in these contexts, it is not known whether racial and ethnic disparities exist in mental healthcare utilization among birthing people who have delivered preterm.

**OBJECTIVE:**

We sought to determine if racial and ethnic disparities exist in postpartum mental healthcare-associated emergency department visits or hospitalizations for birthing people with preterm infants in a large and diverse population.

**STUDY DESIGN:**

This population-based historic cohort study used a sample of Californian live-born infants born between 2011 and 2017 with linked birth certificates and emergency department visit and hospital admission records from the California Statewide Health Planning and Development database. The sample was restricted to preterm infants (<37 weeks’ gestation). Self-reported race and ethnicity groups included Hispanic, non-Hispanic Black, non-Hispanic Asian, non-Hispanic White, and non-Hispanic others. Mental health diagnoses were identified from the International Classification of Diseases Ninth and Tenth revision codes recorded in emergency department and hospital discharge records. Logistic regression analysis was used to estimate the association between mental health-related emergency department visits and rehospitalizations by race or ethnicity compared with non-Hispanic White birthing people and controlling for the following characteristics and health condition covariates: age, parity, previous preterm birth, body mass index, smoking, alcohol use, hypertension, diabetes, previous mental health diagnosis, and prenatal care.

**RESULTS:**

Of 204,539 birthing people who delivered preterm infants in California, 1982 visited the emergency department and 836 were hospitalized in the first year after preterm birth for a mental health-related illness. Black birthing people were more likely to have a mental health-related emergency department visit and hospitalization (risk ratio, 1.8; 95% confidence interval, 1.5–2.0 and risk ratio, 1.9; 95% confidence interval, 1.5–2.3, respectively) within the first postpartum year than White birthing people. Hispanic and Asian birthing people were less likely to have mental health-related emergency department visits (adjusted risk ratio, 0.7; 95% confidence interval, 0.7–0.8 and adjusted risk ratio, 0.2; 95% confidence interval, 0.2–0.3, respectively) and hospitalizations (adjusted risk ratio, 0.6; 95% confidence interval, 0.5–0.7 and adjusted risk ratio, 0.2; 95% confidence interval, 0.1–0.3, respectively). When controlling for birthing people with a previous mental health diagnosis and those without, the disparities remained the same.

**CONCLUSION:**

Racial and ethnic disparities exist in emergency mental healthcare escalation among birthing people who have delivered preterm infants. Our findings highlight a need for further investigation into disparate mental health conditions, exacerbations, access to care, and targeted hospital and legislative policies to prevent emergency mental healthcare escalation and reduce disparities.

## Introduction

A ntenatal and postpartum mental health conditions such as anxiety and depression are prevalent, estimates range from 10% to 25%^1–3^ and have important short- and long-term health consequences for birthing people (a term that recognizes that not all people giving birth identify as women) and their infants.^4–6^ For example, depression has been associated with the development of adverse health outcomes and behaviors during pregnancy, including inadequate nutrition, weight gain, and substance use, and adverse perinatal outcomes like preterm birth (PTB) and low birthweight.^5,7,8^ Postnatally, depression among birthing people has been associated with adverse child hood outcomes including impaired growth, behavior, and cognitive development.^9^ Thus, the prevention, identification, and treatment of mental health conditions during and after pregnancy is critical for both birthing people and infant health. Several risk factors for developing postpartum mental health conditions have been identified, including lower socioeconomic status, history of domestic violence, history of mental health conditions, lack of partner or social support, pregnancy complications, pregnancy loss, and poor infant health.^3,10–12^

Birthing people with preterm infants are at higher risk for anxiety, depression, and stress than birthing people who give birth to term infants.^12^ Birthing people have described unique stressors during neonatal intensive care unit (NICU) hospitalizations including barriers to routine parenting and bonding experiences like holding, protecting, and caring for their infant and have described subsequent posttraumatic stress disorder (PTSD)-like symptoms after NICU hospitalizations.^13^ In addition, giving birth prematurely is a stressful life event that can exacerbate mental health conditions and has been shown to be associated with inpatient mental healthcare utilization postpartum including both emergency department (ED) visits and hospitalizations.^14^

Several studies have shown that non-White birthing people and those with low income are at higher risk for depression and anxiety during and after pregnancy.^2,15–21^ Racial and ethnic disparities have also been described for both outpatient and inpatient postpartum mental healthcare utilization.^22,23^ A study in New Jersey found that Black and Hispanic birthing people were less likely to receive outpatient mental healthcare treatment after delivery, had a longer time from identification to treatment initiation, were less likely to receive continued mental healthcare, and less likely to fill prescriptions for antidepressant medication.^22^ Disparities also exist in potentially avoidable, costly, and morbid inpatient hospitalizations associated with mental health conditions. A study in California found that compared with White birthing people, Black birthing people were at increased risk for receiving postpartum hospital-based mental healthcare.^24^

Racial and ethnic disparities associated with PTB are well described; Black birthing people are more likely to deliver both preterm and low birth-weight infants.^25^ Disparities for preterm infants continue to persist after hospital discharge; Black infants are more likely to be readmitted and die in their first year of life.^26^ Increasingly, structural racism, discrimination, systemic oppression, and social disadvantage are being recognized as sources of chronic stress and poor reproductive health outcomes for Black birthing people.^27–30^

Although previous work has examined disparities in ED and hospital utilization after giving birth to both term and preterm infants,^24^ we specifically chose to focus on preterm infants, because PTB and its complications have also been associated with poor mental health and increased mental healthcare utilization.^14,24^ In addition, although previous work has focused more specifically on postpartum depression,^24^ we elected in this study to examine all mental health conditions including but not limited to mood disorders (depression, bipolar disorder), anxiety related disorders (posttraumatic stress disorder, generalized anxiety disorder), and schizophrenia. Because birthing people of color are a unique population at high risk for PTB, poor infant outcomes, mental health conditions, and mental healthcare utilization, we hypothesized racial and ethnic disparities also exist in mental health-related ED visits and hospitalizations among a population of birthing people who delivered infants preterm.

## Materials and Methods

This population-based historic cohort study analyzed a sample of live-born infants, born between 2011 and 2017 in California, with linked birth certificates, ED visits, and hospital admission records obtained from the California Office of Statewide Health Planning and Development database. The database includes information on infant and birthing people demographics, health conditions, and healthcare utilization up to 1 year postpartum derived from ED and hospital discharge records and birth certificates. Discharge records include diagnosis codes based on the International Classification of Diseases, Ninth and Tenth Revision, Clinical Modification (ICD-9-CM, ICD-10-CM).

### Sample

The sample was restricted to birthing people who delivered singleton, live-born preterm infants between 22 and 36 weeks’ gestation without significant congenital anomalies.The obstetrical age was derived from the gestational age (GA) indicated in the birth certificate based on ultrasound or the last menstrual period.

### Race and ethnicity

Self-reported birthing person race and ethnicity were organized into the following groups: non-Hispanic White (referred to as White), non-Hispanic Black (Black), Hispanic, and non-Hispanic Asian (Asian). “Other” race and ethnicity included American Indian or Alaska Native, Hawaiian or Pacific Islander, other race, >1 race, and those with not stated or unknown race and ethnicity because of sample size constraints among these groups.

### Covariates

We chose covariates known to be associated with PTB and/or mental health conditions during pregnancy, including maternal age at term (<18 years, 18–34 years, >34 years), parity (multiparous or nulliparous), previous PTB, body mass index (BMI) (underweight, normal, overweight, obese, unknown), smoking and/or drug/alcohol abuse during pregnancy, gestational hypertension (HTN), gestational diabetes mellitus (DM), adequate prenatal care,^31^ previous mental health diagnosis, GA, birthweight at GA, infant death, and payer for delivery. Covariate definitions and data sources are listed in Supplemental Table B.

### Mental healthcare utilization

Data on previous mental health conditions and postpartum emergency mental healthcare utilization were obtained from linked hospital discharge records. To identify a birthing person’s mental health diagnoses, relevant ICD-9 and ICD-10 codes were used, including but not limited to mood disorders like depression and bipolar disorder, stress-related disorders like anxiety, behavioral syndrome disorders caused by psychoactive substance use, schizophrenia disorders, and personality disorders (see Supplemental Appendix A). The 4 primary outcomes were mental health-related ED visits and rehospitalizations at 3 months and 1 year postpartum.

### Analysis

We used a logistic regression to test the association of mental health-related ED visits and rehospitalizations with race or ethnicity compared with the referent White birthing person group, chosen as the reference group because of lower rates of mental health utilization in this population based on previous literature, and controlling for birthing people characteristics and health condition covariates previously described by using the following 3 models: model 1 was a crude, unadjusted model. Model 2 adjusted for birthing people characteristics and health condition covariates other than a previous mental health diagnosis. Model 3 adjusted for a previous mental health diagnosis in addition to the covariates in model 2. We stratified preterm infants according to GA groups of <32 weeks and 32 to 36 weeks, populations with significant differences in morbidity and mortality. Methods and protocols for the study were approved by the Committee for the Protection of Human Subjects of the Health and Human Services Agency of the State of California and the institutional review board of the University of California San Francisco.

## Results

**FIGURE 1:**
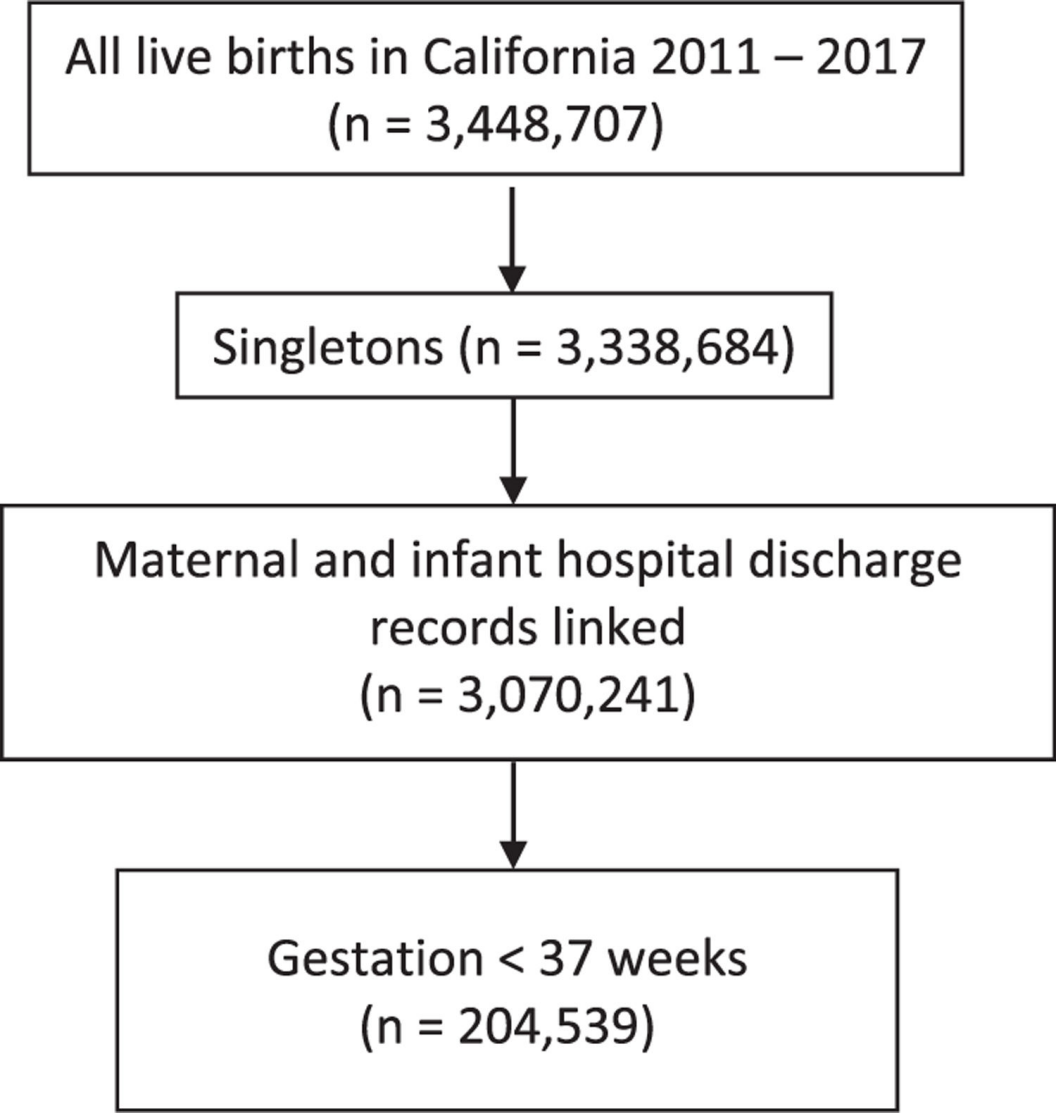
Sample selection Karvonen. Disparities in emergency mental healthcare use after preterm birth. Am J Obstet Gynecol MFM 2021.

Of the 3,448,707 live births in California from 2011 to 2017, 204,539 were preterm singleton infants for whom linked birthing people and infant hospital and discharge records were available (Figure 1).^30^ This sample was 51% Hispanic, 22% non-Hispanic White, 7.3% non-Hispanic Black, 14% non-Hispanic Asian, and 5.5% non-Hispanic other race or ethnicity. Most birthing people were 18 to 34 years old, multiparous, and had adequate prenatal care. Overall, 9.9% of this sample had a previous mental health diagnosis. A total of 12.8% of birthing people delivered infants at <32 weeks GA and 87.2% delivered at 32 to 36 weeks GA. A variety of types of mental health-associated visits were represented within the sample, listed in descending order of frequency as follows: anxiety, mental and behavioral disorders caused by psychoactive substance use, depression, schizophrenia spectrum disorders, other behavioral disorders, and personality disorders (Table 1). Of the birthing people who delivered preterm infants in California, 1089 (0.5%) and 1982 (1.0%) visited the ED for a mental health-related visit within the first 3 months and 1 year after PTB, respectively. Regarding mental health-related hospitalizations, 365 (0.2%) and 836 (0.4%) birthing people were hospitalized in the first 3 months and 1 year after giving birth prematurely (Table 2).

Despite representing only 7.3% of the population in this sample, Black birthing people represented 15.6% of the mental health ED visits in the first year after delivery and 18.1% of the mental health hospitalizations in the first year after delivery. Hispanic and Asian birthing people made up a smaller proportion of ED visits (45.9% and 3.7%, respectively) and hospitalizations (40.3% and 3.4%, respectively) than the representative sample proportion (51.1% and 14.2%, respectively). The proportion of White birthing people with reported ED visits (27.2%) and hospitalizations (29.7%) was more than their sample representation (22.1%) (Table 2).

Compared with White birthing people, Black birthing people were more likely to have a mental health-related ED visit within 3 months and 1 year after birth (crude risk ratio [cRR], 1.7; 95% confidence interval [CI], 1.4–2.0 and cRR, 1.8; 95% CI, 1.5–2.0, respectively). Black birthing people were also more likely to be hospitalized within 3 months and 1 year for a mental health-related illness (cRR 1.9; 95% CI, 1.4–2.6 and cRR, 1.9; 95% CI, 1.5–2.3, respectively). Adjusting for birthing people and infant characteristics, including previous mental healthcare utilization, attenuated some of the excess risk, but Black birthing people continued to be more likely to visit the ED in the first 3 months and 1 year for a mental health-related condition and be hospitalized 1 year postpartum for a mental health-related condition (model 3: adjusted RR [aRR], 1.2; 95% CI, 1.0–1.5; aRR, 1.2; 95% CI, 1.0–1.5; aRR, 1.3; 95% CI, 1.1–1.6, respectively) (Table 2).

Hispanic birthing people were less likely to have a mental health-related ED visit in the first 3 months and 1 year after birth and less likely to have a mental health-related hospitalization in the first 3 months after birth than White birthing people but this likelihood lost statistical significance after controlling for a previous mental health diagnosis (model 2: aRR, 0.7; 95% CI, 0.6–0.9; aRR, 0.8; 95% CI, 0.7–0.9; aRR, 0.7; 95% CI, 0.5–0.9, respectively). Hispanic birthing people were also less likely to have a mental health-related hospitalization 1 year postpartum, which persisted after controlling for covariates (model 3: aRR, 0.8; 95% CI, 0.7–1.0) (Table 2).

Asian birthing people were also significantly less likely to visit the ED in the first 3 months and 1 year for a mental health-related illness than White birthing people, which persisted for all models (model 3: aRR, 0.4; 95% CI, 0.3–0.6; model 3: aRR, 0.4; 95% CI, 0.3 −0.5). Asian birthing people were also less likely to be hospitalized in the first 3 months and 1 year for a mental health-related illness (model 3: aRR, 0.6; 95% CI, 0.3–1.0; model 3: aRR, 0.4; 95% CI, 0.2–0.6) (Table 2).

When stratified by GA groups of <32 and 32 to 36 weeks, disparities were narrowed in the <32 weeks group and remained in 32 to 36 weeks group (Supplemental Table 1 and Supplemental Table 2).

## Discussion

Our findings add to literature describing racial and ethnic disparities in postpartum mental healthcare utilization by highlighting the disparities after preterm delivery. In our study, Black birthing people who have delivered preterm infants are at higher risk for both mental health-related ED visits and rehospitalizations than White birthing people in California. These findings persisted despite controlling for factors known to be associated with mental health conditions including previous mental and physical health conditions and social factors.

A previous study by Chan et al^24^ in which the same database of Californian infants was used also demonstrated racial and ethnic disparities in hospital-based care for postpartum depression. We thought it prudent to expand on this work by investigating whether disparities also exist for birthing people of color who have delivered infants prematurely, because PTB and its complications also have been associated with poor mental health and increased mental healthcare utilization.^14,24^ The Chan et al^24^ study was restricted to birthing people with postpartum depression, whereas our study considered other important mental health conditions, which require a different approach to diagnosis and treatment, faced by birthing people like anxiety, bipolar disorder, schizophrenia, behavioral conditions, personality disorders, and not otherwise specified mental health conditions for a more complete understanding of mental health and severe mental healthcare utilization in this population. In addition, we controlled for infant characteristics like degree of prematurity (<32 vs 32–36 weeks’ gestation) and infant birthweight among preterm infants because prematurity and birthweight have been identified previously as risk factors for mental health conditions.^12^

Families with infants requiring prolonged hospitalization to treat complications of PTB face unique and magnified challenges when compared with those with an uncomplicated postpartum course. PTB may exacerbate postpartum mental health symptoms and existing disparities in postpartum mental healthcare in several ways.^12–14^ For example, preterm infants are at high risk for major preterm comorbidities, unexpected complications requiring procedures and/or surgeries, compromised neurodevelopment impairment, technology dependence and disability, and mortality.^25^ Long hospitalizations and complications of PTB can contribute to financial hardships for families.

Our study also found that when compared with White birthing people, Hispanic and Asian birthing people had a lower risk for mental health-related healthcare utilization after controlling for several covariables, which could reflect underutilization of needed mental health resources or a lesser burden and exacerbation of mental health conditions. Our study cannot make this differentiation and further investigation is necessary. We remain concerned that minority populations may have underdiagnosed and undertreated mental health conditions owing to language barriers, disparate healthcare access, and cultural differences as indicated by previous literature.^22–24^

When stratifying by a GA of <32 weeks, racial disparities narrowed for all groups except for mental health-related hospitalizations for Black birthing people 1 year postpartum, in which case the risk worsened. Disparities persisted in the larger 32- to 36-week GA group. Although there were fewer significant findings in the analysis of infants born <32 weeks, the point estimates were similar to the combined analysis for all preterm infants. We suspect that part of the reason the results became insignificant in this group is that sample sizes for healthcare events became very small. These findings are consistent with previous literature indicating PTB as a risk factor for healthcare utilization^14^ and suggest the need for mental health support for families with preterm infants of all GA.

Although our study does not provide insight into potential interventions to reduce these disparities, we felt it prudent to provide evidence in literature to direct future considerations for investigation and action. Hospital-level interventions that facilitate family-centered care may prevent mental health exacerbations, because improved parent participation during NICU hospitalization and early bonding opportunities are associated with reduced mental health symptoms.^32–35^ Hospital system changes to include free parking, free hospital transportation, and food vouchers for families could alleviate barriers to visitation that structural racism and an unequal distribution of resources, goods, and services secondary to present and historic racism may exacerbate for families of color.^36,37^ Reducing the financial burden by guaranteed and extended maternity leave for hospitalized family members, similar to Canadian policies, could mitigate stressors.^37–39^

Early identification of perinatal and postnatal mental health conditions is paramount for maternal and infant health, and the birth hospitalization presents an opportunity for healthcare access.^40,41^ Routine screening for early intervention of postpartum depression is recommended, however, a recent study found that birthing people of color are less likely to be screened.^42^ Because outpatient referrals often do not lead to sustained outpatient therapy,^43–45^ initiating counseling by mental health providers at the time of birth has been shown to have beneficial results and addresses potential barriers to care such as time availability, stigma, and childcare issues. Recognizing the birth hospitalization as an early intervention access point may lead to improved access, early diagnosis, and treatment.

The American College of Obstetricians and Gynecologists has released a statement that advocates for the optimization of postpartum care with ongoing comprehensive care after birth, including supporting psychological well-being, via regular visits with obstetrical providers. Optimization of postpartum care will require legislative policies that improve the access and quality of community-based mental healthcare and advocate for expanding health insurance coverage postpartum, and interventions that promote social support and reduce financial burden may reduce the impact of PTB on families.^37,38,45^

### Limitations

Although this dataset is large and mental health conditions in pregnancy are common, mental health-related ED visits and hospitalizations are relatively rare events when compared with other reasons for postpartum healthcare utilization, limiting our power to detect significant differences. In addition, because this is a retrospective observational study, we can only deduce association between variables, not causality. Disparate healthcare utilization can reflect disparate access to preventative, outpatient, and/or emergency care or disparate exposures and exacerbations of mental health conditions. Our study is unable to distinguish between the 2 scenarios. This is an important point to distinguish because it impacts where resources would better be spent to reduce these disparities. Important, yet unavailable data included mental health screening rates, records on access to and participation in outpatient mental healthcare, medication access and use, and stressors that may impact mental health including racism, discrimination, food security, social support, major life events, and those lost to follow-up. Similarly, although we suspect that the observed disparate access to resources by race in our dataset is impacted by the historic and present structural racism contributing to unequal access to resources and opportunities for families of color that influences healthcare and outcomes,^30^ we did not have a structural racism metric. Lastly, detailed subcategories to reflect the heterogeneous nature of racial, ethnic, and ancestral groups were not available.

## Conclusion

Racial disparities exist in emergency postpartum mental healthcare utilization for birthing people with preterm infants, with Black birthing people at highest risk for mental health-related hospitalization and ED visits. To achieve health equity for all birthing people, governments and health systems must critically examine the factors that exacerbate this disparity and implement multifaceted interventions.

## Supplementary Material

Appendix A

Appendix B

Table 1

Table 2

## Figures and Tables

**TABLE 1:** Sample characteristics of birthing people and their preterm infants

Characteristics	n	%
Sample	204,539	100
**Race/ethnicity**		
Hispanic	104,451	51.1
**Non-Hispanic**		
White	45,354	22.2
Black	14,853	7.3
Asian	28,698	14
Other	11,183	5.5
**Parent age at delivery (y)**		
<18	4006	2
18–34	150,584	73.6
>34	49,930	24.4
Missing	19	0
**Parent education (y)**		
<12	39,977	19.5
12	52,538	25.7
>12	102,333	50
Missing	9691	4.7
**Parity**		
Nulliparous	78,367	38.3
Multiparous	125,917	61.6
Missing	255	0.1
**Adequacy of prenatal care** ^a^		
Adequate plus/adequate	156,476	76.5
Intermediate	16,910	8.3
Inadequate	23,803	11.6
Missing	7350	3.6
**Payer for delivery**		
Private	90,342	44.2
Public	101,879	49.8
Other	12,318	6
Previous preterm birth	8794	4.3
**BMI**		
Underweight	8334	4.1
Normal	83,064	40.6
Overweight	50,731	24.8
Obese	51,556	25.2
Missing	10,854	5.3
Smoking	10,615	5.2
Drug/alcohol use	10,246	5
Hypertension	49,518	24.2
Diabetes	38,236	18.7
Mental health disorders in pregnancy	20,185	9.9
**Gestational age**		
<32	26,169	12.8
32–36	178,370	87.2
Birthweight		
SGA	20,486	10
AGA	164,320	80.3
LGA	18,669	9.1
Infant death	6505	3.2
**Any mental**		
Mental Health Condition	2085	1.0
Mental and behavioral disorders owing to psychoactive substance use	544	0.3
Schizophrenia, schizotypal and delusional disorders	228	0.1
Mood (affective) disorders	685	0.33
Depression	457	0.2
Bipolar disorder	270	0.1
Neurotic, stress-related and somatoform disorders	1257	0.6
Anxiety	963	0.5
Behavioral syndromes associated with physiological disturbances and physical factors	182	0.1
Personality disorders	7	<0.1

*AGA,* appropriate for gestational age; *BMI,* body mass index; *LGA,* large for gestational age; *SGA,* small for gestational age.

a From Kotelchuck.^31^

Karvonen. Disparities in emergency mental healthcare use after preterm birth. Am J Obstet Gynecol MFM 2021.

**TABLE 2:** Risk ratios of mental healthcare utilization by race and ethnicity, <37 weeks

Variables	No mental healthcare utilization within 1 y postpartum	Mental health-related ED visit w/in 3 mo postpartum	Mental health-related ED visit w/in 1 y postpartum	Mental health-related hospitalization within 3 mo postpartum	Mental health-related hospitalization within 1 y postpartum	
Sample		202,245	1089	1982	365	836
Race and ethnicity						
Hispanic	n (%)	103,504(51.1)	479 (44.0)	909 (45.9)	145(38.7)	337 (40.3)
	Model 1 RR (95% CI)		0.6 (0.6–0.7)^a^	0.7 (0.7—0.8)^a^	0.6 (0.4—0.7)^a^	0.6 (0.5—0.7)^a^
	Model 2 RR (95% CI)		0.7 (0.6–0.9)^a^	0.8 (0.7—0.9)^a^	0.7 (0.5—0.9)^a^	0.7 (0.6—0.8)^a^
	Model 3 RR (95% CI)		0.9 (0.7–1.0)	0.9 (0.8—1.0)	0.8 (0.6—1.1)	0.8 (0.7—1.0)^a^
Black	n (%)	14,524(7.2)	179 (16.4)	309 (15.6)	69 (18.9)	151 (18.1)
	Model 1 RR (95% CI)		1.7 (1.4—2.0)^a^	1.8 (1.5—2.0)^a^	1.9 (1.4—2.6)^a^	1.9 (1.5—2.3)^a^
	Model 2 RR (95% CI)		1.2 (1.0—1.5)^a^	1.3 (1.1 —1.5)^a^	1.4 (1.0—1.9)	1.3 (1.1—1.6)^a^
	Model 3 RR (95% CI)		1.2 (1.0—1.5)^a^	1.2 (1.0—1.5)^a^	1.4 (1.0—1.9)	1.3 (1.1—1.6)^a^
Asian	n (%)	28,615(14.1)	37 (3.4)	73 (3.7)	16(4.4)	28 (3.4)
	Model 1 RR (95% CI)		0.2 (0.1 —0.3)^a^	0.2 (0.2—0.3)^a^	0.2 (0.1 —0.4)^a^	0.2 (0.1 —0.3)^a^
	Model 2 RR (95% CI)		0.3 (0.2—0.4)^a^	0.3 (0.3—0.4)^a^	0.4 (0.2—0.7)^a^	0.3 (0.2—0.4)^a^
	Model 3 RR (95% CI)		0.4 (0.3—0.6)^a^	0.4 (0.3—0.5)^a^	0.6 (0.3—1.0)^a^	0.4 (0.2—0.6)^a^
Other	n (%)	11,022 (5.4)	72 (6.6)	152(7.7)	26(7.1)	72 (8.6)
	Model 1 RR (95% CI)		0.9 (0.7—1.2)	1.1 (1.0—1.4)	1.0 (0.6—1.5)	1.2 (0.9—1.5)
	Model 2 RR (95% CI)		0.8 (0.6—1.0)^a^	1.0 (0.8—1.2)	0.8 (0.5—1.2)	1.0 (0.8—1.3)
	Model 3 RR (95% CI)		0.8 (0.6—1.0)	1.0 (0.8—1.2)	0.8 (0.5—1.3)	1.0 (0.8—1.3)
White non-Hispanic (reference)	n (%)	44,789 (22.1)	322 (29.6)	539 (27.2)	109 (29.9)	248 (29.7)

Model 1: unadjusted.

Model 2 adjusted for: Maternal age a term, parity, previous preterm birth, BMI, smoking during pregnancy, drug/alcohol abuse during. pregnancy, hypertension, diabetes, adequate prenatal care, gestational age (continuous), birthweight for GA, infant death, payer for delivery.

Model 3 adjusted for: prior mental health diagnosis in addition to model 2 variables.

*BMI,* body mass index; *Cl,* confidence interval; *ED,* emergency department; *GA,* gestational age; *RR,* relative risk..

a Statistical significance *P*<.05..

Karvonen. Disparities in emergency mental healthcare use after preterm birth. Am J Obstet Gynecol MFM 2021.

## AJOG MFM at a Glance

### Why was this study conducted?

Birthing people of color experience higher rates of postpartum mental health conditions, preterm birth, poor infant outcomes, and mental healthcare utilization. Preterm birth-associated hospitalizations are an additional contributor to poor mental health during the postpartum period. It is not known whether racial and ethnic disparities exist in emergency mental healthcare utilization by birthing people who deliver preterm infants.

### Key findings

In this large and diverse population-based cohort, racial and ethnic disparities existed in mental healthcare utilization after preterm birth, with Black birthing people more likely to have a mental health-related emergency department visit or hospitalization 1 year postpartum (relative risk [RR], 1.8; 95% confidence interval [CI], 1.5–2.0; RR, 1.9; 95% CI, 1.5–2.3) compared with White birthing people.

### What does this add to what is known?

This study highlights the racial and ethnic disparities in emergency mental healthcare escalation in birthing people who have delivered preterm infants. Targeted interventions in high-risk groups may promote equitable outcomes.

## References

[1] Gavin NI, Gaynes BN, Lohr KN, Meltzer-Brody S, Gartlehner G, Swinson T. Perinatal depression: a systematic review of prevalence and incidence. Obstet Gynecol 2005;106: 1071–83. 16260528 10.1097/01.AOG.0000183597.31630.db

[2] Melville JL, Gavin A, Guo Y, Fan MY, Katon WJ. Depressive disorders during pregnancy: prevalence and risk factors in a large urban sample. Obstet Gynecol 2010;116:1064–70. 20966690 10.1097/AOG.0b013e3181f60b0a PMC3068619

[3] Biaggi A, Conroy S, Pawlby S, Pariante CM. Identifying the women at risk of antenatal anxiety and depression: a systematic review. J Affect Disord 2016;191:62–77. 26650969 10.1016/j.jad.2015.11.014 PMC4879174

[4] Slomian J, Honvo G, Emonts P, Reginster JY, Bruyére O. Consequences of maternal postpartum depression: a systematic review of maternal and infant outcomes. Womens Health (Lond) 2019;15:1745506519844044. 31035856 10.1177/1745506519844044 PMC6492376

[5] Marcus SM, Heringhausen JE. Depression in childbearing women: when depression complicates pregnancy. Prim Care 2009;36:151–65. 19231607 10.1016/j.pop.2008.10.011 PMC2680254

[6] Räisänen S, Lehto SM, Nielsen HS, Gissler M, Kramer MR, Heinonen S. Risk factors for and perinatal outcomes of major depression during pregnancy: a population-based analysis during 2002–2010 in Finland. BMJ Open 2014;4:e004883. 10.1136/bmjopen-2014-004883 PMC4244456 25398675

[7] Zuckerman B, Amaro H, Bauchner H, Cabral H. Depressive symptoms during pregnancy: relationship to poor health behaviors. Am J Obstet Gynecol 1989;160:1107–11. 2729387 10.1016/0002-9378(89)90170-1

[8] Grote NK, Bridge JA, Gavin AR, Melville JL, Iyengar S, Katon WJ. A meta-analysis of depression during pregnancy and the risk of preterm birth, low birth weight, and intrauterine growth restriction. Arch Gen Psychiatry 2010;67:1012–24. 20921117 10.1001/archgenpsychiatry.2010.111 PMC3025772

[9] Grace SL, Evindar A, Stewart DE. The effect of postpartum depression on child cognitive development and behavior: a review and critical analysis of the literature. Arch Womens Ment Health 2003;6:263–74. 14628179 10.1007/s00737-003-0024-6

[10] Stein A, Malmberg LE, Sylva K, Barnes J, Leach P, team FCCC**. The influence of maternal depression, caregiving, and socioeconomic status in the post-natal year on children’s language development. Child Care Health Dev 2008;34:603–12. 18549438 10.1111/j.1365-2214.2008.00837.x

[11] Witt WP, Wisk LE, Cheng ER, Poor prepregnancy and antepartum mental health predicts postpartum mental health problems among US women: a nationally representative population-based study. Womens Health Issues 2011;21:304–13. 21349740 10.1016/j.whi.2011.01.002 PMC3126903

[12] Vigod SN, Villegas L, Dennis CL, Ross LE. Prevalence and risk factors for postpartum depression among women with preterm and low-birth-weight infants: a systematic review. BJOG 2010;117:540–50. 20121831 10.1111/j.1471-0528.2009.02493.x

[13] Shaw RJ, Deblois T, Ikuta L, Ginzburg K, Fleisher B, Koopman C. Acute stress disorder among parents of infants in the neonatal intensive care nursery. Psychosomatics 2006; 47:206–12. 16684937 10.1176/appi.psy.47.3.206

[14] Calthorpe LM, Baer RJ, Chambers BD, The association between preterm birth and postpartum mental healthcare utilization among California birthing people. Am J Obstet Gynecol MFM 2021;3:100380. 33932629 10.1016/j.ajogmf.2021.100380

[15] Fellenzer JL, Cibula DA. Intendedness of pregnancy and other predictive factors for symptoms of prenatal depression in a population-based study. Matern Child Health J 2014;18:2426–36. 24752314 10.1007/s10995-014-1481-4

[16] Faisal-Cury A, Rossi Menezes P. Prevalence of anxiety and depression during pregnancy in a private setting sample. Arch Womens Ment Health 2007;10:25–32. 17187166 10.1007/s00737-006-0164-6

[17] Jesse DE, Swanson MS. Risks and resources associated with antepartum risk for depression among rural southern women. Nurs Res 2007;56:378–86. 18004184 10.1097/01.NNR.0000299856.98170.19

[18] Rich-Edwards JW, Kleinman K, Abrams A, Sociodemographic predictors of antenatal and postpartum depressive symptoms among women in a medical group practice. J Epidemiol Community Health 2006;60:221–7. 16476752 10.1136/jech.2005.039370 PMC2465548

[19] Canady RB, Bullen BL, Holzman C, Broman C, Tian Y. Discrimination and symptoms of depression in pregnancy among African American and white women. Womens Health Issues 2008;18:292–300. 18590883 10.1016/j.whi.2008.04.003 PMC2872142

[20] Orr ST, Blazer DG, James SA. Racial disparities in elevated prenatal depressive symptoms among black and white women in eastern North Carolina. Ann Epidemiol 2006;16:463–8. 16257228 10.1016/j.annepidem.2005.08.004

[21] Sakala C, Declercq ER, Turon JM, Corry MP. Listening to mothers in California: a population-based survey of women’s childbearing experiences, full survey report [internet]; 2018. Available at: www.chcf.org/wp-content/uploads/2018/09/ListeningMothersCAFullSurveyReport2018.pdf, https://bp.chcf.org/wp-content/uploads/2018/09/ListeningMothersCAFullSurveyReport2018.pdf. DC: WA.

[22] Kozhimannil KB, Trinacty CM, Busch AB, Huskamp HA, Adams AS. Racial and ethnic disparities in postpartum depression care among low-income women. Psychiatr Serv 2011;62:619–25. 21632730 10.1176/appi.ps.62.6.619 PMC3733216

[23] Seplowitz R, Miller H, Ostermeyer B, Sangi-Haghpeykar H, Silver E, Kunik ME. Utilization of psychiatric services by postpartum women in a predominantly minority, low-socioeconomic-status, urban population. Community Ment Health J 2015;51:275–80. 25535052 10.1007/s10597-014-9808-6

[24] Chan AL, Guo N, Popat R, Racial and ethnic disparities in hospital-based care associated with postpartum depression. J Racial Ethn Health Disparities 2021;8:220–9. 32474833 10.1007/s40615-020-00774-y

[25] Martin JA, Hamilton BE, Osterman MJK, Driscoll AK. Births: final data for 2018. Natl Vital Stat Rep 2019;68:1–47. 32501202

[26] Karvonen KL, Baer RJ, Rogers EE, Racial and ethnic disparities in outcomes through 1 year of life in infants born prematurely: a population based study in California. J Perinatol 2021;41:220–31. 33514879 10.1038/s41372-021-00919-9

[27] McLemore MR, Altman MR, Cooper N, Williams S, Rand L, Franck L. Health care experiences of pregnant, birthing and postnatal women of color at risk for preterm birth. Soc Sci Med 2018;201:127–35. 29494846 10.1016/j.socscimed.2018.02.013

[28] Chambers BD, Erausquin JT, Tanner AE, Nichols TR, Brown-Jeffy S. Testing the association between traditional and novel indicators of county-level structural racism and birth outcomes among black and white women. J Racial Ethn Health Disparities 2018;5:966–77. 29218496 10.1007/s40615-017-0444-z

[29] Chambers BD, Baer RJ, McLemore MR, Jelliffe-Pawlowski LL. Using index of concentration at the extremes as indicators of structural racism to evaluate the association with preterm birth and infant mortality-California, 2011–2012. J Urban Health 2019;96:159–70. 29869317 10.1007/s11524-018-0272-4 PMC6458187

[30] Sutton S, Kubisch A, Susi G. Fulbright-Anderson K. Structural racism and community building. The Aspen Institute Roundtable on Community Change; 2004. Available at https://www.aspeninstitute.org/wp-content/uploads/files/content/docs/rcc/aspen_structural_racism2.pdf. Accessed April 1st 2021.

[31] Kotelchuck M An evaluation of the Kessner Adequacy of Prenatal Care Index and a proposed Adequacy of Prenatal Care Utilization Index. Am J Public Health 1994;84:1414–20. 8092364 10.2105/ajph.84.9.1414 PMC1615177

[32] Sinha B, Sommerfelt H, Ashorn P, Effect of community-initiated kangaroo mother care on postpartum depressive symptoms and stress among mothers of low-birth-weight infants: a randomized clinical trial. JAMA Netw Open 2021;4:e216040. 33885776 10.1001/jamanetworkopen.2021.6040 PMC8063066

[33] Gangi S, Dente D, Bacchio E, Giampietro S, Terrin G, De Curtis M. Posttraumatic stress disorder in parents of premature birth neonates. Procedia Soc Behav Sci 2013;82:882–5.

[34] Welch MG, Halperin MS, Austin J, Depression and anxiety symptoms of mothers of preterm infants are decreased at 4 months corrected age with Family Nurture Intervention in the NICU. Arch Womens Ment Health 2016;19:51–61. 25724391 10.1007/s00737-015-0502-7

[35] Lee HC, Martin-Anderson S, Dudley RA. Clinician perspectives on barriers to and opportunities for skin-to-skin contact for premature infants in neonatal intensive care units. Breastfeed Med 2012;7:79–84. 22011130 10.1089/bfm.2011.0004 PMC3317520

[36] Lakshmanan A, Agni M, Lieu T, The impact of preterm birth <37 weeks on parents and families: a cross-sectional study in the 2 years after discharge from the neonatal intensive care unit. Health Qual Life Outcomes 2017;15:38. 28209168 10.1186/s12955-017-0602-3 PMC5312577

[37] Greenfield JC, Klawetter S. Parental leave policy as a strategy to improve outcomes among premature infants. Health Soc Work 2016;41:17–23. 26946882 10.1093/hsw/hlv079

[38] Government of Canada. EI caregiving benefits. 2021. Available at: https://www.canada.ca/en/services/benefits/ei/caregiving.html. Accessed April 27, 2021.

[39] Hoffman C, Dunn DM, Njoroge WFM. Impact of postpartum mental illness upon infant development. Curr Psychiatry Rep 2017;19: 100. 29105008 10.1007/s11920-017-0857-8

[40] Hynan MT, Steinberg Z, Baker L, Recommendations for mental health professionals in the NICU. J Perinatol 2015;35(Suppl1): S14–8. 26597800 10.1038/jp.2015.144 PMC4660044

[41] Sidebottom A, Vacquier M, LaRusso E, Erickson D, Hardeman R. Perinatal depression screening practices in a large health system: identifying current state and assessing opportunities to provide more equitable care. Arch Womens Ment Health 2021;24: 133–44. 32372299 10.1007/s00737-020-01035-x PMC7929950

[42] Kallem S, Matone M, Boyd RC, Guevara JP. Mothers’ mental health care use after screening for postpartum depression at well-child visits. Acad Pediatr 2019;19: 652–8. 30496869 10.1016/j.acap.2018.11.013

[43] Shaw RJ, St John N, Lilo EA, Prevention of traumatic stress in mothers with preterm infants: a randomized controlled trial. Pediatrics 2013;132:e886–94. 23999956 10.1542/peds.2013-1331 PMC3784295

[44] Goodman JH. Women’s attitudes, preferences, and perceived barriers to treatment for perinatal depression. Birth 2009; 36:60–9. 19278385 10.1111/j.1523-536X.2008.00296.x

[45] ACOG Committee Opinion No. 736: optimizing postpartum care. Obstet Gynecol 2018;131:e140–50. 29683911 10.1097/AOG.0000000000002633

