# Supplementary material for: Racial disparities in emergency mental healthcare utilization among birthing people with preterm infants
Source: Am J Obstet Gynecol MFM. Author manuscript; Available in PMC 2022 Mar 22. (PMC8939261; doi:10.1016/j.ajogmf.2021.100546)
Supplement: Appendix A [file NIHMS1785141-supplement-Appendix_A.docx]

S**upplemental Appendix A. ICD-9 and ICD-10 codes for Mental Health Diagnoses**

| **Type of mental health disorder for visit** | **ICD-10** | **ICD-9** |
| --- | --- | --- |
| Mental and behavioral disorders due to psychoactive substance use | F1 | 303.x, 304.x, 305.x |
| Schizophrenia, schizotypal and delusional disorders | F2 | 295.x |
| Mood [affective] disorders | F3 | 296.x, 311 |
| Depression | F32, F33, F34, F38, F39 | 311, 296.2, 296.3 |
| Bipolar disorder | F30, F31 | 296.0, 296.1, 296.4, 296.5, 296.6, 296.7, 296.8, 296.9 |
| Neurotic, stress-related and somatoform disorders | F4 | 297.x, 298.x, 299.x, 300.x, 306.x, 308.x, 309.x |
| Anxiety | F41 | 300.x |
| Behavioral syndromes associated with physiological disturbances and physical factors | F5 | 307.x |
| Eating disorders | F50 | 307.1, 307.5 |
| Nonorganic sleep disorders | F51 | 307.4 |
| Disorders of adult personality and behavior | F60, F61, F62, F63, F67, F68, F69 | 301.x, |
