# Supplementary material for: Racial disparities in emergency mental healthcare utilization among birthing people with preterm infants
Source: Am J Obstet Gynecol MFM. Author manuscript; Available in PMC 2022 Mar 22. (PMC8939261; doi:10.1016/j.ajogmf.2021.100546)
Supplement: Appendix B [file NIHMS1785141-supplement-Appendix_B.docx]

**Supplementary Appendix B. Appendix of Variables**

| **Variable** | **Subcategory** | **Source** | **Definition** | **Dichotomous vs. Ordinal vs. Nominal** |
| --- | --- | --- | --- | --- |
| Male or Female |  | Birth certificate record |  | dichotomous |
| GA |  | Birth certificate record | Best obstetrical age: ultrasound or last menstrual period | ordinal |
|  | <32 weeks |  |  |  |
|  | 32-36 weeks |  |  |  |
| Birthweight |  | Birth certificate record | birthweight, sex, and gestational age were used to correct for implausible gestational age^1^ | ordinal |
|  | SGA |  |  |  |
|  | AGA |  |  |  |
|  | LGA |  |  |  |
|  | Missing |  |  |  |
| Maternal Age |  | Birth certificate record |  | ordinal |
|  | <18 years |  |  |  |
|  | 18-34 years |  |  |  |
|  | >34 years |  |  |  |
|  | Missing |  | valid range limited to 13 - 55 |  |
| Maternal Education |  | Birth certificate record |  | ordinal |
|  | <12 years |  |  |  |
|  | 12 years |  |  |  |
|  | >12 years |  |  |  |
|  | Missing |  |  |  |
| Maternal BMI |  | Birth certificate record | Calculated from pre-pregnancy birth weight and height | ordinal |
|  | Under weight |  | < 18.5 kg/m^2^ |  |
|  | Normal weight |  | 18.5 - < 25 kg/m^2^ |  |
|  | Over weight |  | 25 - < 30 kg/m^2^ |  |
|  | Obese |  | ≥30 kg/m^2^ |  |
|  | Missing |  |  |  |
| Maternal smoking during pregnancy |  | Birth certificate record and hospital discharge records | ICD9 649.0, 305.1, ICD10 P04.2, Z72.0, F17.2 | dichotomous |
| Maternal alcohol use |  | Hospital discharge records | ICD9 303, 305.0 ICD10 F10 | dichotomous |
| Maternal illicit drug use |  | Hospital discharge records | ICD9 304, 305.2, 305.3, 305.4, 305.5, 305.6, 305.7, 305.8, 305.9, ICD10 F11, F12, F13, F14, F15, F16, F18, F19 | dichotomous |
| Insurance |  | Birth certificate record |  | nominal |
|  | Private |  |  |  |
|  | Public |  |  |  |
|  | Other |  |  |  |
| WIC participation |  | Birth certificate record |  | nominal |
|  | Yes |  |  |  |
|  | No |  |  |  |
|  | Unknown |  |  |  |
| Maternal HTN |  | Birth certificate record and hospital discharge records | ICD9 642, 760.0, ICD10 P00.0, O1 | dichotomous |
| Maternal DM |  | Birth certificate record and hospital discharge records | ICD9 648.0, 648.8, ICD10 P70.0, P70.1, O24.0, O24.1, O24.2, O24.3, O24.4, O24.9 | dichotomous |
| Maternal Mental Illness |  | Hospital discharge records |  | dichotomous |
| Maternal Prenatal Care |  | Birth certificate record | Created from month of entry into care, number of prenatal visits, and gestation at birth^2^ | ordinal |
|  | Adequate plus/adequate |  |  |  |
|  | Inadequate |  |  |  |
|  | Missing |  |  |  |
| ^1^ Talge NM, Mudd LM, Sikorskii A, and Basso O. United States birth weight reference corrected for implausible gestational age estimates. *Pediatrics* 2014; 133(5):844-53. | | | | |
| ^2^Kotelchuck M. An evaluation of the Kessner Adequacy of Prenatal Care Index and a proposed Adequacy of Prenatal Care Utilization Index. *Am J Public Health*. 1994;84(9):1414-20. | | | | |
