# Supplementary material for: Racial disparities in emergency mental healthcare utilization among birthing people with preterm infants
Source: Am J Obstet Gynecol MFM. Author manuscript; Available in PMC 2022 Mar 22. (PMC8939261; doi:10.1016/j.ajogmf.2021.100546)
Supplement: Table 1 [file NIHMS1785141-supplement-Table_1.docx]

| **Supplemental Table 1. Risk ratios of mental health care utilization by race and ethnicity, < 32 weeks** | | | | | | |
| --- | --- | --- | --- | --- | --- | --- |
|  |  | No mental health care utilization within 1 year postpartum | Mental health-related ED visit w/in 3 months postpartum | Mental health-related ED visit w/in 1 year postpartum | Mental health-related hospitalization within 3 months postpartum | Mental health-related hospitalization within 1 year postpartum |
| Sample |  | 25,836 | 184 | 317 | 65 | 133 |
| Race and Ethnicity |  |  |  |  |  |  |
| Hispanic | n (%) | 13,110 (50.7) | 91 (49.5) | 154 (48.6) | 26 (40.0) | 57 (42.9) |
|  | Model 1 RR (95% CI) |  | 0.8 (0.6, 1.2) | 0.9 (0.6, 1.1) | 0.6 (0.3, 1.1) | 0.7 (0.5, 1.1) |
|  | Model 2 RR (95% CI) |  | 1.1 (0.7, 1.6) | 1.1 (0.8, 1.5) | 0.9 (0.4, 1.8) | 1.0 (0.6, 1.7) |
|  | Model 3 RR (95% CI) |  | 1.2 (0.8, 1.9) | 1.2 (0.9, 1.7) | 1.1 (0.5, 2.1) | 1.2 (0.8, 2.0) |
| Black | n (%) | 2,938 (11.4) | 36 (19.6) | 66 (20.8) | 16 (24.6) | 35 (26.3) |
|  | Model 1 RR (95% CI) |  | 1.4 (0.9, 2.2) | **1.6 (1.2, 2.3)** | 1.6 (0.8, 3.1) | **1.9 (1.2, 3.1)** |
|  | Model 2 RR (95% CI) |  | 1.2 (0.7, 1.8) | 1.4 (1.0, 2.0) | 1.2 (0.6, 2.5) | 1.6 (1.0, 2.7) |
|  | Model 3 RR (95% CI) |  | 1.2 (0.7, 1.9) | 1.2 (0.7, 1.9) | 1.2 (0.6, 2.3) | 1.6 (0.9, 2.6) |
| Asian | n (%) | 3,258 (12.5) | ^a^ | 9 (2.8) | ^a^ | ^a^ |
|  | Model 1 RR (95% CI) |  | **--** | **0.2 (0.1, 0.4)** | **--** | **--** |
|  | Model 2 RR (95% CI) |  | **--** | **0.4 (0.2, 0.8)** | **--** | **--** |
|  | Model 3 RR (95% CI) |  | **--** | **0.5 (0.2, 0.9)** | **--** | **--** |
| Other | n (%) | 1,641 (6.4) | 11 (6.0) | 21 (6.6) | ^a^ | 8 (6.0) |
|  | Model 1 RR (95% CI) |  | 0.8 (0.4, 1.5) | 0.9 (0.6, 1.5) | **--** | 0.8 (0.4, 1.7) |
|  | Model 2 RR (95% CI) |  | 0.8 (0.4, 1.5) | 0.9 (0.5, 1.5) | **--** | 0.8 (0.4, 1.8) |
|  | Model 3 RR (95% CI) |  | 0.8 (0.4, 1.5) | 0.9 (0.6, 1.5) | **--** | 0.8 (0.4, 1.9) |
| White non-Hispanic (reference) | n (%) | 4,898 (19.0) | 42 (22.8) | 67 (21.1) | 17 (26.2) | 30 (22.6) |
| Model 1: unadjusted | |  |  |  |  |  |
| Model 2 adjusted for: Maternal age a term, parity, previous preterm birth, BMI, smoking during pregnancy, drug/alcohol abuse during pregnancy, hypertension, diabetes, adequate prenatal care, gestational age (continuous), birthweight for GA, infant death, payer for delivery | | | | | | |
| Model 3 adjusted for: prior mental health diagnosis in addition to model 2 variables | | | | | | |
| bold typing indicates statistical significance p<0.05 | | | |  |  |  |
| ^a^n < 5, RR not calculated. | | | |  |  |  |
