# Supplementary material for: Racial disparities in emergency mental healthcare utilization among birthing people with preterm infants
Source: Am J Obstet Gynecol MFM. Author manuscript; Available in PMC 2022 Mar 22. (PMC8939261; doi:10.1016/j.ajogmf.2021.100546)
Supplement: Table 2 [file NIHMS1785141-supplement-Table_2.docx]

| **Supplemental Table 2. Risk ratios of mental health care utilization by race and ethnicity, 32 – 36 weeks** | | | | | |  |
| --- | --- | --- | --- | --- | --- | --- |
|  |  | No MH care utilization within 1 year postpartum | MH-related ED visit w/in 3 months postpartum | MH-related ED visit w/in 1 year postpartum | MH-related hospitalization within 3 months postpartum | MH-related hospitalization within 1 year postpartum |
| Sample |  | 176,618 | 905 | 1,665 | 300 | 703 |
| Race and Ethnicity |  |  |  |  |  |  |
| Hispanic | n (%) | 90,394 (51.2) | 388 (42.9) | 755 (45.4) | 119 (39.7) | 280 (39.8) |
|  | Model 1 RR (95% CI) |  | **0.6 (0.5, 0.7)** | **0.7 (0.6, 0.8)** | **0.6 (0.4, 0.8)** | **0.6 (0.5, 0.7)** |
|  | Model 2 RR (95% CI) |  | **0.7 (0.6, 0.8)** | **0.8 (0.7, 0.9)** | **0.7 (0.5, 0.9)** | **0.7 (0.5, 0.8)** |
|  | Model 3 RR (95% CI) |  | **0.8 (0.7, 0.9)** | **0.9 (0.8, 1.0)** | 0.8 (0.6, 1.1) | **0.8 (0.6, 0.9)** |
| Black | n (%) | 11,845 (6.6) | 143 (15.8) | 243 (14.6) | 53 (17.7) | 116 (16.5) |
|  | Model 1 RR (95% CI) |  | **1.7 (1.4, 2.1)** | **1.8 (1.5, 2.1)** | **2.0 (1.4, 2.8)** | **1.8 (1.5, 2.3)** |
|  | Model 2 RR (95% CI) |  | 1.2 (1.0, 1.5) | **1.3 (1.1, 1.5)** | 1.4 (1.0, 2.0) | **1.3 (1.0, 1.6)** |
|  | Model 3 RR (95% CI) |  | **1.2 (1.0, 1.5)** | **1.2 (1.0, 1.5)** | 1.4 (1.0, 2.0) | **1.3 (1.0, 1.6)** |
| Asian | n (%) | 25,366 (14.4) | 33 (3.7) | 64 (3.8) | 14 (4.7) | 25 (3.6) |
|  | Model 1 RR (95% CI) |  | **0.2 (0.1, 0.3)** | **0.2 (0.2, 0.3)** | **0.2 (0.1, 0.4)** | **0.2 (0.1, 0.3)** |
|  | Model 2 RR (95% CI) |  | **0.3 (0.2, 0.4)** | **0.3 (0.3, 0.4)** | **0.4 (0.2, 0.7)** | **0.3 (0.2, 0.4)** |
|  | Model 3 RR (95% CI) |  | **0.4 (0.3, 0.6)** | **0.4 (0.3, 0.5)** | 0.6 (0.3, 1.1) | **0.4 (0.2, 0.6)** |
| Other | n (%) | 9,521 (5.3) | 61 (6.7) | 131 (7.9) | 22 (7.3) | 64 (9.1) |
|  | Model 1 RR (95% CI) |  | 0.9 (0.7, 1.2) | 1.2 (1.0, 1.4) | 1.0 (0.6, 1.6) | 1.2 (0.9, 1.6) |
|  | Model 2 RR (95% CI) |  | 0.8 (0.6, 1.0) | 1.0 (0.8, 1.2) | 0.8 (0.5, 1.3) | 1.0 (0.8, 1.3) |
|  | Model 3 RR (95% CI) |  | 0.8 (0.6, 1.0) | 1.0 (0.8, 1.2) | 0.8 (0.5, 1.3) | 1.1 (0.8, 1.4) |
| White non-Hispanic (reference) | n (%) | 39,891 (22.6) | 280 (30.9) | 472 (28.4) | 92 (30.7) | 218 (31.0) |
| Model 1: unadjusted | |  |  |  |  |  |
| Model 2 adjusted for: Maternal age a term, parity, previous preterm birth, BMI, smoking during pregnancy, drug/alcohol abuse during pregnancy, hypertension, diabetes, adequate prenatal care, gestational age (continuous), birthweight for GA, infant death, payer for delivery | | | | | | |
| Model 3 adjusted for: prior mental health diagnosis in addition to model 2 variables | | | | | | |
| bold typing indicates statistical significance p<0.05 | | | |  |  |  |
